# Supplementary material for: Survival of cyanobacteria and mitigation of Fe(II) toxicity effects in a silica-rich Archean ocean
Source: Nat Commun. 2026 Feb 21;17:1987. doi: 10.1038/s41467-026-69826-x (PMC12932812; doi:10.1038/s41467-026-69826-x)
Supplement: Supplementary file 1 — Supplementary information [file 41467_2026_69826_MOESM1_ESM.pdf]

**SUPPORTING INFORMATION**

# Survival of cyanobacteria and mitigation of Fe(II) toxicity effects in a silica-rich Archean ocean

Carolin L. Dreher[1], Olaf A. Cirpka[2], Manuel Schad[3,4], Kurt O. Konhauser[3], Andreas Kappler[1,5*]

[1]Geomicrobiology, Department of Geosciences, University of Tuebingen, Tuebingen, Germany.

[2]Hydrogeology, Department of Geosciences, University of Tuebingen, Tuebingen, Germany.

[3]Department of Earth and Atmospheric Sciences, University of Alberta, Edmonton, Alberta, Canada.

[4]Now: GFZ Helmholtz Centre for Geosciences, Section of Geomicrobiology, Potsdam, Germany.

[5]Cluster of Excellence EXC 2124, Controlling Microbes to Fight Infection, University of Tuebingen, Germany.

Corresponding author:

[*]Andreas Kappler (andreas.kappler@uni-tuebingen.de)

***For submission to Nature Communications***

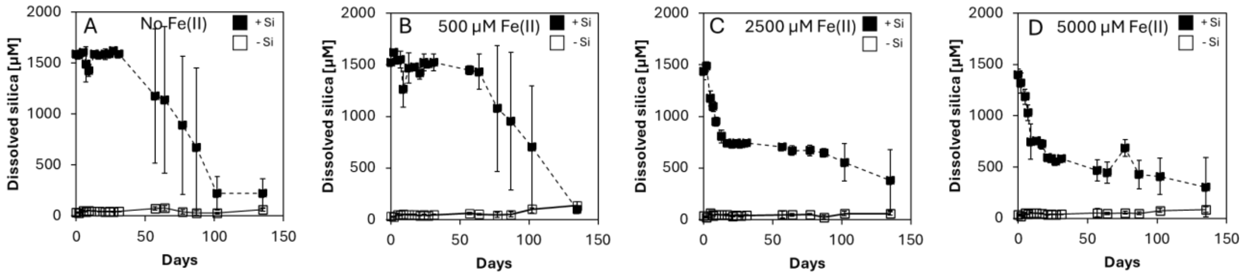


*Fig. S1:* **Dissolved silica data of Fe(II) oxidation experiments containing *Synechococcus* PCC 7002.** *Panel A shows the results of setups without amendment of Fe(II)$_{(aq)}$. Panel B shows the results of setups with amendment of 500 µM Fe(II)$_{(aq)}$, panel C with 2500 µM Fe(II)$_{(aq)}$, and panel D with 5000 µM Fe(II)$_{(aq)}$. Filled symbols indicate setups with 2200 µM silica ('high silica'), empty symbols indicate setups without silica ('no silica'). Dissolved silica (black) is plotted as average values from three triplicates with the standard deviation as error bars of the experiments.*

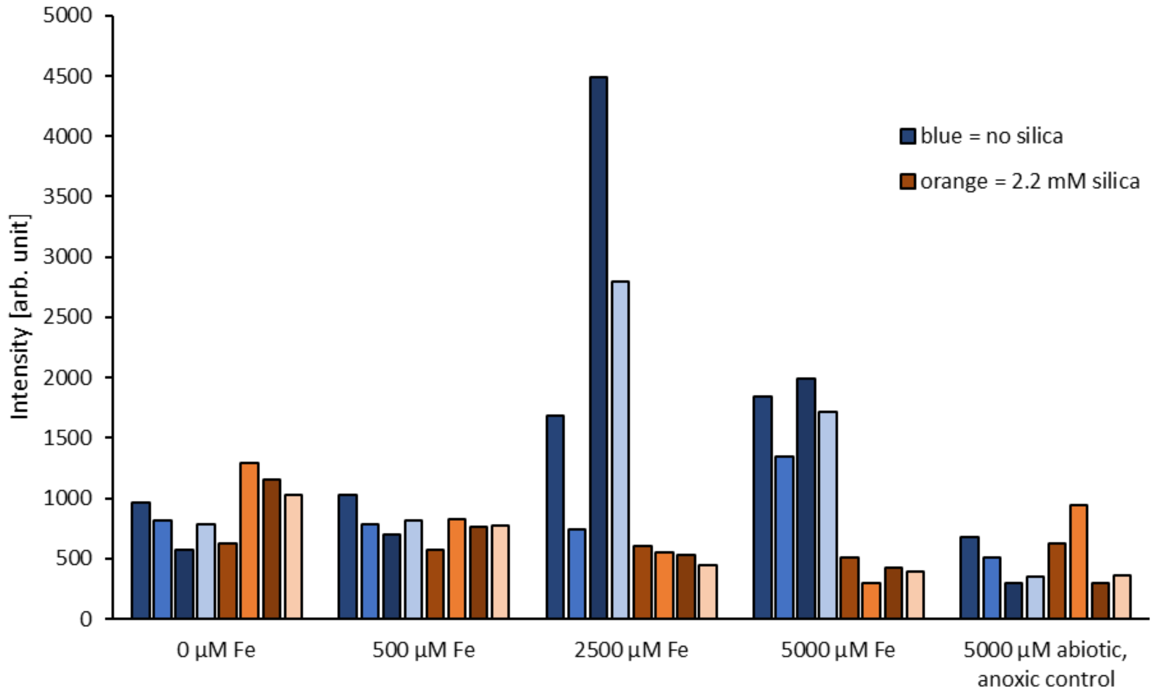


*Fig. S2:* **Impact of silica on ROS formation at different iron concentrations.** *ROS fluorescence signals of experimental setups containing strain PCC 7002 cells, either 0, 500, 2500 or 5000 µM Fe(II)$_{(aq)}$ in the absence (blue) and presence of 2200 µM SiO$_{2(aq)}$ (orange).*

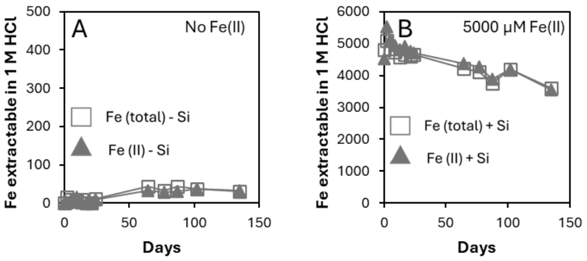


*Fig. S3:* **Sterile controls of the Fe(II) oxidation experiments.** *Panel A shows the results of setups without amendment of Fe(II)$_{(aq)}$ or Si. Panel B shows the results of setups with amendment of 5000 µM Fe(II)$_{(aq)}$ and 2200 µM Si.*

*Table S1:* **Geochemical data of ROS incubations.** *Measured Fe(tot) and dissolved silica concentrations of the ROS measurement. The controls are anoxic, abiotic setups with and without silica in the presence of 5000 µM Fe(II)$_{aq}$. The 'initial' concentrations represent the theoretical added concentrations, the 'final' concentrations' the measured concentrations at the end of the incubations.*

| Initial Fe(II)$_{aq}$ [µM] | Initial silica$_{aq}$ [µM] | Final Fe(tot) [µM] | Final silica$_{aq}$ [µM] |
|---|---|---|---|
| 0 | 0 | 0.0 ± 0 | 11.1 ± 19 |
| 500 | 0 | 566.8 ± 63 | 24.2 ± 36 |
| 2500 | 0 | 2703.0 ± 119 | 2.6 ± 2 |
| 5000 | 0 | 5325.1 ± 148 | 0.0 ± 0 |
| 5000 control | 0 | 5151.9 ± 122 | 0.0 ± 0 |
| 0 | 2200 | 0.0 ± 0 | 811.0 ± 186 |
| 500 | 2200 | 566.4 ± 31 | 392.4 ± 24 |
| 2500 | 2200 | 2382.8 ± 125 | 549.4 ± 123 |
| 5000 | 2200 | 5003.0 ± 243 | 317.1 ± 170 |
| 5000 control | 2200 | 4631.4 ± 193 | 203.5 ± 54 |

**Numerical Model of the Lab Experiments**

We assume that the batch reactors are perfectly mixed and that gas exchange between the aqueous solution and the head space is in equilibrium. The photosynthesis rate $r_{photo}$ [μM$_{DO}$/d] is proportional to the mean radiation $\bar{I}$ [W/m$^2$] within the reactor and the concentration $c_{bio}^{active}$ [cells/mL] of the active cyanobacteria, and may be inhibited by elevated ferrous-iron concentrations:

$$r_{photo} = \bar{I} k_{photo}^{*} c_{bio}^{alive} f_{inh}(c_{Fe(II)}), \tag{1}$$

where $k_{photo}^{*}$ [μM$_{DO}$/d×mL/cells×m$^2$/W] is the efficiency of photosynthesis. In the course of the experiment, the suspension containing cyanobacteria is getting increasingly turbid leading to adsorption of radiation. We account for this integrating Lambert-Beer's law of absorption over depth:

$$\bar{I} = \frac{1}{Z}\int_0^Z I(z)dz = \frac{1}{Z}\int_0^Z I_0 \exp(-\lambda c_{bio}^{tot} z)\, dz = \frac{I_0}{Z\lambda c_{bio}^{tot}}(1 - \exp(-\lambda c_{bio}^{tot} Z)) \tag{2}$$

where $z$ [m] is the depth coordinate, $Z$ [m] is the total depth, $I_0$ [W/m$^2$] is the radiation at the surface $c_{bio}^{tot}$ [cells/mL] is the concentration of cyanobacteria both active and inactive ($c_{bio}^{tot} = c_{bio}^{active} + c_{bio}^{inactive}$ with $c_{bio}^{inactive}$ [cells/mL] being the concentration of inactive cyanobacteria), and $\lambda$ [mL/cells/m] is the adsorption coefficient. We define the following variables:

$$k_{photo} = I_0 k_{photo}^{*} \tag{3}$$

$$c_{bio}^{ref} = \frac{1}{Z\lambda} \tag{4}$$

where $k_{photo}$ [μM$_{DO}$/d×mL/cells = nmol$_{DO}$/d/cell] is the photosynthesis coefficient at the surface radiation, and $c_{bio}^{ref}$ [cells/mL] is a reference cell concentration for light absorption. Then the photosynthesis rate becomes:

$$r_{photo} = k_{photo} c_{bio}^{active} \frac{c_{bio}^{ref}}{c_{bio}^{tot}} \left(1 - \exp\left(-\frac{c_{bio}^{tot}}{c_{bio}^{ref}}\right)\right) f_{inh}(c_{Fe(II)}) \tag{5}$$

which depends on the concentrations of both active and inactive cyanobacteria.

Swanner and collegues[1] determined the following inhibition law:

$$f_{inh}(c_{Fe(II)}) = \left(\frac{K_{inh}}{K_{inh} + c_{Fe(II)}}\right)^{n_{inh}} \tag{6}$$

where $K_{inh}$ = 1 $\mu M_{Fe(II)}$ is an inhibition concentration, and $n_{inh}$ [-] is an exponent. With the small value of $K_{inh}$ the inhibition law is practically a power law:

$$\lim_{c_{Fe(II)}/K_{inh} \to \infty} f_{inh}(c_{Fe(II)}) = c_{Fe(II)}^{-n_{inh}} \tag{7}$$

Dissolved oxygen is reduced by ferrous iron, for which assume bimolecular kinetics:

$$r_{FeOx} = \gamma_{FeOx} c_{DO} c_{Fe(II)} \tag{8}$$

where $r_{FeOx}$ [$\mu M_{DO}$/d] is the oxygen reduction rate by iron oxidation, $\gamma_{FeOx}$ [1/d/$\mu M_{Fe(II)}$] is the corresponding rate coefficient, and $c_{Fe(II)}$ [$\mu M_{Fe(II)}$] is the concentration of ferrous iron in solution.

In our model, oxygen undergoes equilibrium partitioning between the aqueous solution in the head space, leading to retardation of the rate of change of concentration in aqueous solution with a retardation factor $R$ [-] defined by:

$$R = 1 + \frac{V_g}{V_w} k_H^{cc} \tag{9}$$

where $V_w$ [L] and $V_g$ [L] are the volumes of water and gas, respectively, and $k_H^{cc} \approx 42$ is the dimensionless Henry's law constant of dissolved oxygen at room temperature. Living cyanobacteria grow proportional to the photosynthesis rate with a yield coefficient $Y$ [cells/mL/$\mu M_{DO}$ = cells/$nmol_{DO}$], and they decay according to a first-law with decay coefficient $k_{dec}$ [1/d].

The system of ordinary differential equations is now defined as:

$$R\frac{dc_{DO}}{dt} = r_{photo} - r_{FeOx} \tag{10}$$

$$\frac{dc_{bio}^{active}}{dt} = Yr_{photo} - k_{dec}c_{bio}^{active} \tag{11}$$

$$\frac{dc_{bio}^{inactive}}{dt} = k_{dec}c_{bio}^{active} \tag{12}$$

$$\frac{dc_{Fe(II)}}{dt} = -4r_{FeOx} \tag{13}$$

subject to initial conditions. $c_{bio}^{active}(0)$ was assumed to be the average cell concentration at time zero, $c_{bio}^{inactive}(0)$ was assumed to be zero, $c_{DO}(0)$ and $c_{Fe(II)}(0)$ were also set to the average concentrations measured at time zero. The system of ordinary differential equations was solved using Gear's method as implemented in the matlab function ode15s [2].

We could calculate $R$ as 68.2, whereas the coefficients $k_{photo}$, $c_{bio}^{ref}$, $Y$, $k_{dec}$, and $\gamma_{FeOx}$ were dertemined by jointly fitting the experiments without silica and iron, with 2mM silica but without iron, and with 2mM silica and 0.5mM ferrous iron. The underlying assumption was that the fitted coefficients don't depend on the ferrous-iron concentrations in the presence of dissolved silica. We fitted the logarithms of the parameters using the trust-region reflective least square method implemented in lsqnonlin of matlab's optimization toolbox. Because Fe(II) concentrations were at late times appeared erratic, we considered only those values at the begin of simulations where a clear trend was observable. We performed to model fits, one accounting for inhibition of photosynthesis by elevated ferrous-iron concentrations, and the other neglecting it.

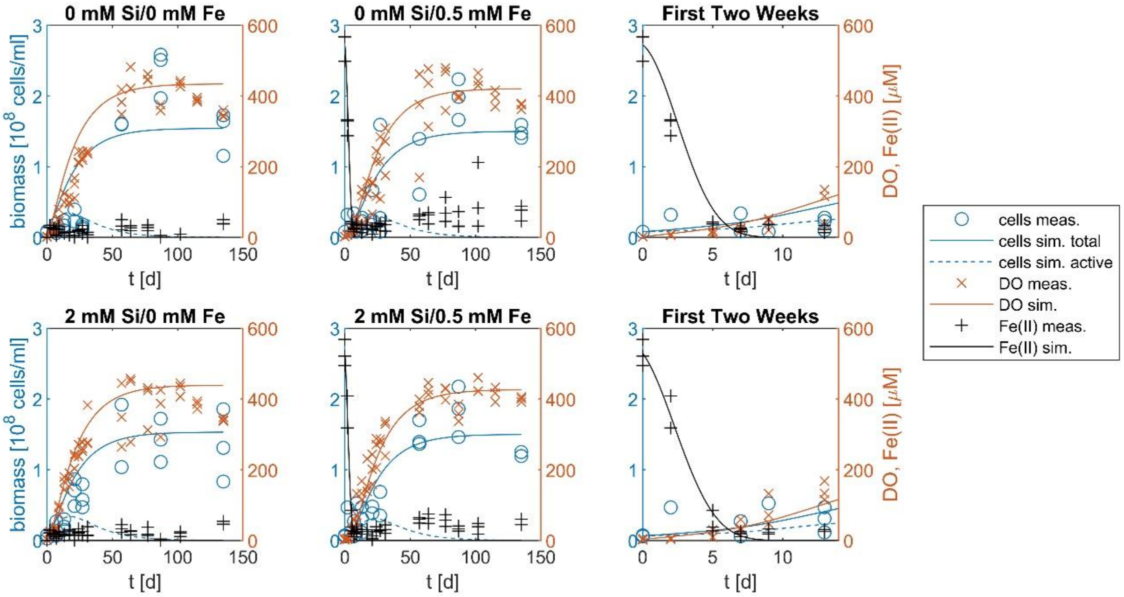


*Fig. S4:* **Modelled lab data of the experimental setups containing 500 µM iron without silica (top) and with 2200 µM silica (bottom) considering photosynthesis inhibition**. *Blue ('cell sim. total'),orange ('DO sim.') and black ('Fe(II) sim.') lines are the modeled total cell densities and oxygen concentrations, respectively, the blue circles ('cells meas.'), the orange crosses ('DO meas.') and the black crosses ('Fe(II) meas.') show the associated raw data points. The bright blue dashed line ('cells sim. active') shows the calculated concentration of active cells.*

Fig. S4 shows the model fit of the experiments for the model variant with photosynthesis inhibition by ferrous iron. The light absorption makes the photosynthesis per living cell less effective at late times so that biomass deactivation/decay prevails. This in turn leads to the decline of oxygen production by photosynthesis. Table S2 lists the associated fitted coefficients and their factors of uncertainty. The latter are computed by applying linear uncertainty propagation of the log-parameters, leading to the standard deviations of all log-parameters. The factors of uncertainty are the exponentials of these standard deviations.

*Table S2:* **Fitted coefficients for the model accounting for photosynthesis inhibition** *by ferrous iron.*

| variable | fitted value | | factor of uncertainty |
|---|---|---|---|
| $k_{photo}$ | $8.92\times10^{-5}$ | $\mathrm{nmol_{DO}/d/cell}$ | 1.20 |
| $Y$ | 4940 | $\mathrm{cells/nmol_{DO}}$ | 1.05 |
| $c_{bio}^{ref}$ | $1.98\times10^{7}$ | cells/mL | 1.66 |

| | | | |
|---|---|---|---|
| $k_{dec}$ | 0.109 | /d | 1.37 |
| $\gamma_{FeOx}$ | $7.32\times10^{-3}$ | /d/$\mu M_{Fe(II)}$ | 1.23 |

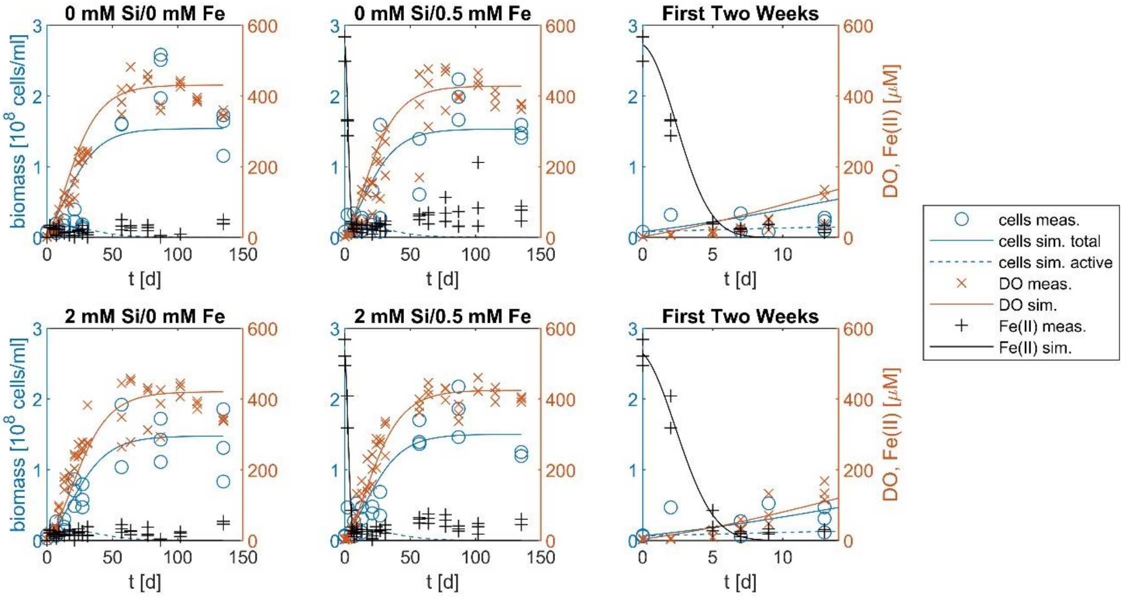


*Fig. S5:* **Modelled lab data of the experimental setups containing 500 µM iron without silica (top) and with 2200 µM silica (bottom) neglecting photosynthesis inhibition**. *Blue ('cell sim.total'),orange ('DO sim.') and black ('Fe(II) sim.') lines are the modeled total cell densities and oxygen concentrations, respectively, the blue circles ('cells meas.'), the orange crosses ('DO meas.') and the black crosses ('FeII) meas.') show the associated raw data points. The bright blue dashed line ('cells sim. active') shows the calculated concentration of active cells.*

*Table S3:* **Fitted coefficients for the model neglecting for photosynthesis inhibition** *by ferrous iron.*

| variable | fitted value | | factor of uncertainty |
|---|---|---|---|
| $k_{photo}$ | $6.54\times10^{-5}$ | $nmol_{DO}$/d/cell | 1.47 |
| $Y$ | 4980 | cells/$nmol_{DO}$ | 1.05 |
| $c_{bio}^{ref}$ | $1.13\times10^{8}$ | cells/mL | 4.44 |
| $k_{dec}$ | 0.243 | /d | 2.35 |
| $\gamma_{FeOx}$ | $5.17\times10^{-3}$ | /d/$\mu M_{Fe(II)}$ | 1.36 |

Fig. S5 shows the fit of the model neglecting photosynthesis inhibition by ferrous iron, and Table S3 lists the associated parameters. While the model fit is of similar quality, the parameters are considerably more uncertain and correlated. The fitted inactivation/decay coefficient appears fairly high, which might be seen as a compensation mechanism for not having any inhibition at early times when ferrous iron concentrations are still very high.

**Transfer to the Archean Ocean**

The following description is based on [1]. We assume steady-state 1-D vertical reactive transport of ferrous iron and oxygen in the ocean:

$$-v\frac{\partial c_{DO}}{\partial z}-\frac{\partial}{\partial z}\left(D(z)\frac{\partial c_{DO}}{\partial z}\right)=r_{photo}(z)-r_{FeOx} \tag{14}$$

$$-v\frac{\partial c_{Fe(II)}}{\partial z}-\frac{\partial}{\partial z}\left(D(z)\frac{\partial c_{Fe(II)}}{\partial z}\right)=-4r_{FeOx} \tag{15}$$

subject to the following boundary conditions:

$$c_{DO}(z_{\max})=0, \qquad c_{Fe(II)}(z_{\max})=c_{Fe(II)}^{\max} \tag{16}$$

$$c_{DO}(0)=0, \qquad \left.\frac{\partial c_{Fe(II)}}{\partial z}\right|_{z=0}=0 \tag{17}$$

where $z$ [m] is the depth coordinate, $v$ [m/s] is a constant upwelling velocity, $D(z)$ [m$^2$/s] denotes the vertical mixing coefficient, $z_{\max}$ [m] is the maximum depth considered, and $c_{Fe(II)}^{\max}$ [µM] is the ferrous-iron concentration at depth. The top boundary condition for dissolved oxygen implies an atmosphere without any oxygen.

We assume a uniform abundance of cyanobacteria and a uniform turbidity in the phototrophic zone leading to an exponential decline of the photosynthesis rate $r_{photo}(z)$ with depth:

$$r_{photo}(z)=r_{photo}^{\max}\exp\left(-\frac{z}{z_{photo}}\right)f_{inh}(c_{Fe(II)}) \tag{18}$$

where $r_{photo}^{\max}$ [µM$_{DO}$/d] is the maximum photosynthesis rate occurring at the ocean surface, and $z_{photo}$ [m] is the light-penetration depth. If the physiological state of the Archean cyanobacteria was comparable to those in the lab experiment, $r_{photo}^{\max}$ could be computed by:

$$r_{photo}^{\max}=k_{photo}c_{bio}^{ocean}\frac{I_0^{ocean}}{I_0^{lab}} \tag{19}$$

where $c_{bio}^{ocean}$ [cells/mL] is the cell density of active cyanobacteria in the phototrophic zone of the Archean ocean, whereas $I_0^{ocean}$ [W/m$^2$] and $I_0^{lab}$ [W/m$^2$] are the mean radiations at the surface of the Archean ocean and at the top of the lab experiment, respectively.

The turbulent mixing coefficient $D(z)$ in the ocean is assumed to follow a double-linear trend:

$$D(z) = f(x) = \begin{cases} D_{top} + \frac{z}{z_{picno}}\left(D_{picno} - D_{top}\right), & z < z_{picno} \\ D_{picno} + \left(z - z_{picno}\right)\left.\frac{dD}{dz}\right|_{z \geq z_{picno}}, & z \geq z_{picno} \end{cases} \tag{20}$$

where $z_{picno}$ [m] is the depth of the picnocline, where the mass density of water is the highest, $D_{top}$ [m$^2$/s] and $D_{picno}$ [m$^2$/s] are the mixing coefficients at the ocean surface and at the pycnocline, whereas $\left.\frac{dD}{dz}\right|_{z \geq z_{picno}}$ [m/s] is the vertical gradient of the mixing coefficient below the picnocline.

Equations 14 & 15 are discretized in space using a Finite Volume scheme with a resolution of 0.25m. Because of the nonlinear interactions, we simulate transient transport with constant coefficients for 1000 years when steady state is reached. The resulting system of ordinary differential equations was using Gear's method as implemented in the matlab function ode15s [2].

*Table S4:* **Parameters for the steady-state advective-diffusive transport model.** *Parameters are based on the study of Swanner and colleagues[1]. The parameters with asterix (*) were added for our own calculations.*

| | Value | Reference |
|---|---|---|
| Water depth | 500 m | [3] |
| Photosynthetic depth | 100 m | [4] |
| Depth of pycnocline | 50 m | [5], Black sea |
| *Eddy-diffusion coefficient | 1 x $10^{-5}$ $m^2$ $s^{-1}$ at top | [5], Black sea, calculated from vertical profile of iron and manganese in the black sea |
| Temperature | 22°C | [5] |
| pH | 8 | [6] |
| Upwelling rate | Variable (4, 95 and 473 m $yr^{-1}$) | [7] |
| $O_2$ release rate | 4% of modern value | |
| Solar radiation at water surface* | 170 W/$m^2$ | Modern value |
| Cyanobacterial cell density* | $10^5$ - $10^6$ cells/mL | [8,9] |
| Iron concentration* | ~ 500 µM | [10,11] |

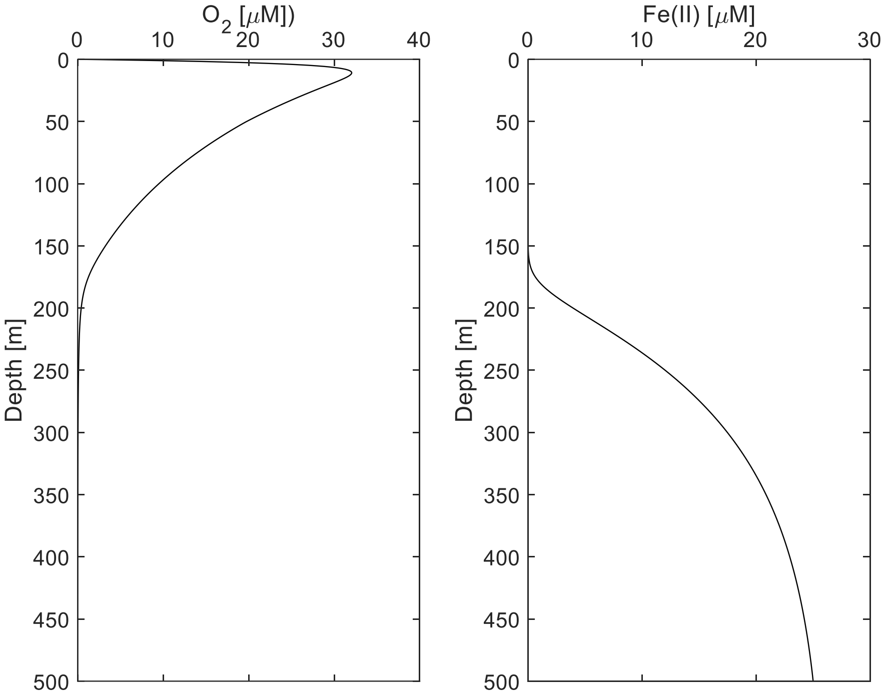


*Fig. S6:* **Modelled oxygen (left) and Fe(II) (right) concentration in the ancient water column** *for an upwelling velocity of 95 m/yr and for 25 µM Fe(II) based on the photosynthetic rate (0.112 µM/day) obtained from our lab experiments containing 529 µM Fe(II).*

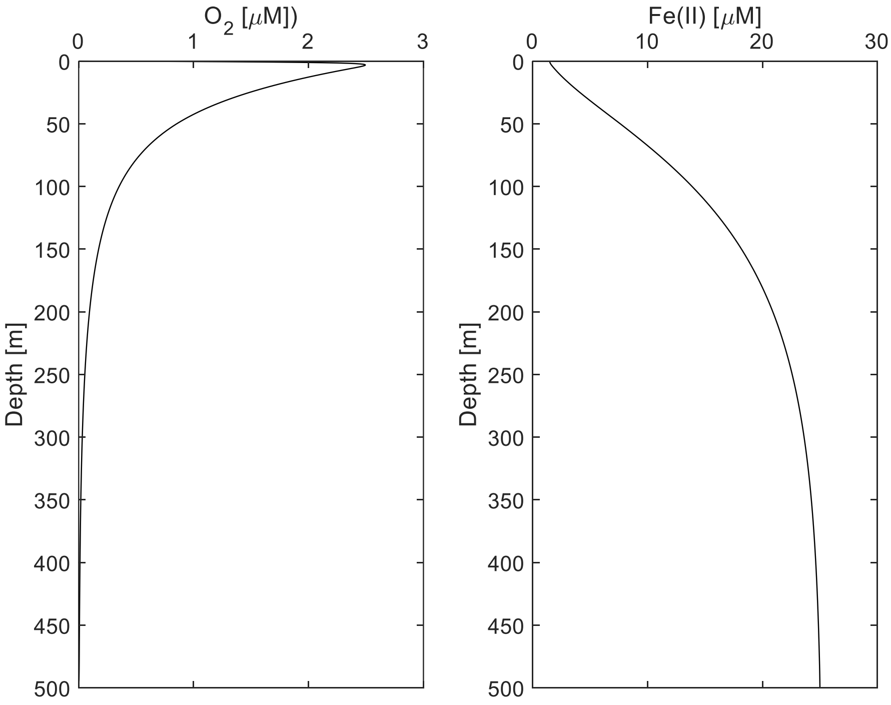


*Fig. S7:* **Modelled oxygen (left) and Fe(II) (right) concentration in the ancient water column** *for an upwelling velocity of 473 m/yr and for 25 µM Fe(II) based on the photosynthetic rate (0.112 µM/day) obtained from our lab experiments containing 529 µM Fe(II).*

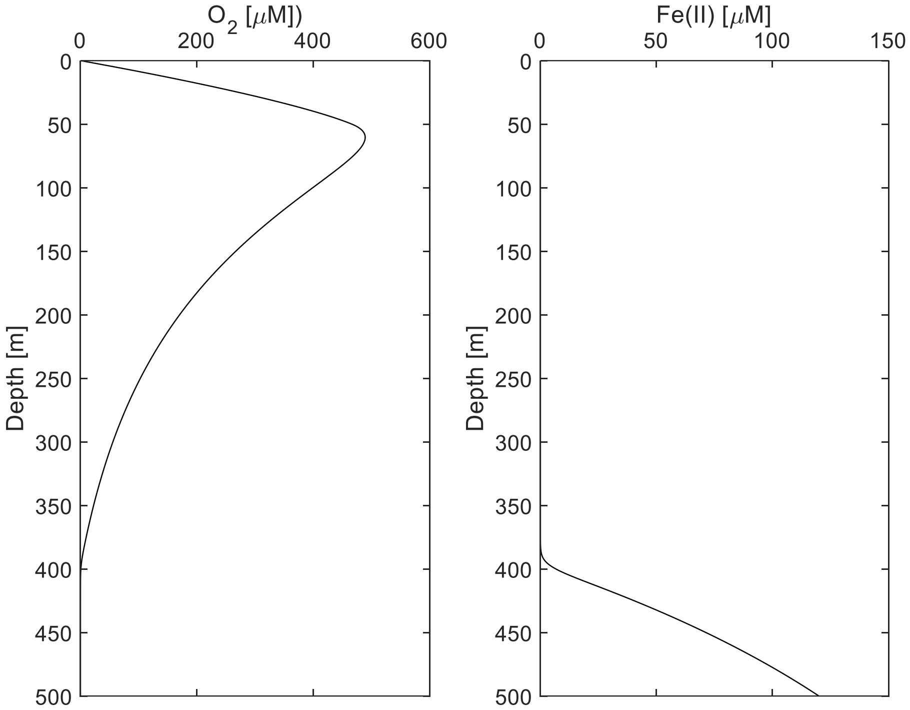


*Fig. S8:* **Modelled oxygen (left) and Fe(II) (right) concentration in the ancient water column** *for an upwelling velocity of 4 m/yr and for 120 µM Fe(II) based on the photosynthetic rate (0.112 µM/day) obtained from our lab experiments containing 529 µM Fe(II).*

1. Swanner, E. D. *et al.* Modulation of oxygen production in Archean oceans by episodes of Fe(II) toxicity. *Nat. Geosci.* **8**, 126–130 (2015).
2. Shampine, L. F. & Reichelt, M. W. The MATLAB ODE Suite. *SIAM J. SCI. COMPUT.* **18**, 1–22 (1997).
3. Beukes, N. J. & Gutzmer, J. Origin and paleoenvironmental significance of major iron formations at the Archean-Paleoproterozoic boundary. *Rev. Econ. Geol.* **15**, 5–47 (2008).
4. Flombaum, P. *et al.* Present and future global distributions of the marine Cyanobacteria Prochlorococcus and Synechococcus. *Proc. Natl. Acad. Sci.* **110**, 9824–9829 (2013).
5. Lewis, B. L. & Landing, W. M. The biogeochemistry of manganese and iron in the Black Sea. *Deep Sea Res. Part Oceanogr. Res. Pap.* **38**, S773–S803 (1991).
6. Grotzinger, J. P. & Kasting, J. F. New Constraints on Precambrian Ocean Composition. *J. Geol.* (1993) doi:10.1086/648218.
7. Trabucho Alexandre, J. *et al.* The mid-Cretaceous North Atlantic nutrient trap: Black shales and OAEs. *Paleoceanography* **25**, (2010).
8. Konhauser, K. O. *et al.* Could bacteria have formed the Precambrian banded iron formations? *Geology* **30**, 1079 (2002).
9. Konhauser, K. O. *et al.* Phytoplankton contributions to the trace-element composition of Precambrian banded iron formations. *GSA Bull.* **130**, 941–951 (2018).
10. Holland, H. D. The oceans: a possible source of iron in iron-formations. *Econ. Geol.* **68**, 1169–1172 (1973).
11. Morris, R. C. Genetic modelling for banded iron-formation of the Hamersley Group, Pilbara Craton, Western Australia. *Precambrian Res.* **60**, 243–286 (1993).
